# Supplementary material for: Effectiveness evaluation of China’s water resource tax reform pilot and path optimization from the perspective of policy field
Source: PLoS One. 2024 Mar 28;19(3):e0301031. doi: 10.1371/journal.pone.0301031 (PMC10977700; doi:10.1371/journal.pone.0301031)
Supplement: Attachment — Submitted filename: Response to Reviewers0130.docx [file pone.0301031.s003.docx]

**Modification instructions**

Dear editors,

Thanks for providing us with this great opportunity to submit a revised version of our manuscript. We appreciate the detailed and constructive comments provided by the reviewers. We have carefully revised the manuscript by incorporating all the suggestions by the reviewers.

We hope this revised manuscript has addressed your concerns, and look forward to hearing from you.

Sincerely,

First author: Mingyi YANG

Second author: Muqi ZHOU

Corresponding author: Conglin ZHANG

**Encl. Responses to the comments from Reviewers.**

**Reply to Reviewer #2**

We appreciate your comments on this article and hope that our response adequately addresses all of your concerns. In the remainder of this letter, we will respond to each of your comments and will revise them accordingly.

To facilitate this discussion, we first transcribe your comments in italics types and then present our responses to the comments.

**Comment 1: *Fig. 1: Add Latitude and Longitude to the map.***

**Response 1:** We apologize for the lack of normality in Figure 1 without latitude and longitude. We appreciate your comments and have added latitude and longitude to figure 1.

**Correction1:**

3.2 Data Source and Case Selection

In July 2016, Hebei Province became the first pilot area for water resources tax reform in China. In November 2017, the Ministry of Finance of China, China State Administration of Taxation, and the Ministry of Water Resources of China jointly issued the *Implementation Measures for Expanding the Pilot Program of Water Resources Tax Reform*, which expanded the scope of the pilot to 9 provinces (autonomous regions and municipalities) including Beijing, Tianjin, Shanxi, Inner Mongolia, Henan, Shandong, Sichuan, Ningxia and Shaanxi ^[4]^. This study selects “1+9” water resources tax reform pilot areas as the main body of the case study, the location of these case study areas is shown in Figure 1.

Figure 1 The location of “1+9” water resources tax reform pilot areas

**Comment 2: *Table 4: header and columns are not matched.***

**Response 2:** We appreciate your careful review. The original title of Table 4 was indeed problematic, and we have revised the formatting of Table 4 with reference to papers of the same type. In addition, to increase the match between the Conclusions section and the empirical operationalization steps, we moved Table 4 to section 4.2.

**Correction 2:**

4.2 Construction of Truth Tables and Analysis Results of Necessary Conditions

Prior to the necessity analysis, the Truth table is constructed based on the results of variable assignments (Table 4), in which each row represents a logically possible configuration, or combination of factors/conditions associated with a given outcome ^[44]^. The presence (1) or absence (0) of conditions is specified for each row.

Table 3 Truth Table

| Case | C_1_ | C_2_ | C_3_ | C_4_ | C_5_ | C_6_ | C_7_ | C_8_ | C_9_ | C_10_ |
| --- | --- | --- | --- | --- | --- | --- | --- | --- | --- | --- |
| **Beijing** | 1 | 1 | 1 | 1 | 1 | 1 | 1 | 1 | 1 | 1 |
| **Tianjin** | 0 | 1 | 0 | 1 | 1 | 1 | 0 | 0 | 0 | 0 |
| **Hebei** | 1 | 0 | 0 | 1 | 0 | 0 | 1 | 0 | 0 | 0 |
| **Shanxi** | 1 | 1 | 1 | 0 | 1 | 1 | 1 | 0 | 0 | 1 |
| **Inner Mongolia** | 1 | 0 | 1 | 1 | 1 | 1 | 1 | 0 | 0 | 1 |
| **Shandong** | 0 | 0 | 0 | 1 | 1 | 1 | 1 | 1 | 1 | 0 |
| **Henan** | 0 | 1 | 0 | 1 | 0 | 1 | 1 | 0 | 1 | 0 |
| **Sichua** | 1 | 0 | 0 | 1 | 0 | 1 | 0 | 1 | 1 | 0 |
| **Shaanxi** | 1 | 1 | 1 | 0 | 1 | 1 | 0 | 0 | 0 | 1 |
| **Ningxia** | 0 | 0 | 0 | 1 | 1 | 1 | 0 | 1 | 1 | 0 |

Table 4 reports results of our necessity analysis. No condition met or exceeded the consistency score threshold of 0.90, indicating that none of them can be explained as a necessary condition affecting the effect of water resource tax policy, and the influence of each condition variable on the policy effect has strong interdependence, and different condition combinations have different impacts on the policy effect. On this basis, the condition combinations are further tested and analyzed.

Table 4 Analysis of conditions for necessity consistency and coverage

| **Condition** | **Necessity Consistency** | **Necessity Coverage** |
| --- | --- | --- |
| Water Resource Endowment | 0.73 | 0.87 |
| Industrial Structure | 0.54 | 0.84 |
| Technological Level | 0.57 | 0.81 |
| Tax Object Design | 0.75 | 0.84 |
| Tax Rate Design | 0.72 | 0.84 |
| Tax-free Situation Design | 0.67 | 0.81 |
| Tax Collection and Management Model | 0.57 | 0.81 |
| Tax Collection Situation | 0.79 | 0.86 |
| Regional Water-saving Effect | 0.71 | 0.87 |
| Supervision of Water Resource Tax | 0.69 | 0.80 |

**Comment 3:** ***the conclusion section is still long.***

**Response 3:** We appreciate your feedback and agree with your suggestions. We have streamlined the conclusion section, reducing what was originally over 600 words to around 400 words, to ensure that the conclusion section provides a high level summary of the entire research.

**Correction 3:**

**6. Conclusion**

This paper constructs a general theoretical and methodological framework for public policy evaluation, which is "the decision and decomposition of policy goals–the selection and implementation of policy goals–policy supervision and security." In addition, public policy evaluation and natural resource measurement methods are combined to construct an evaluation index system from the perspective of the water resource tax policy field to evaluate the effect of the policy, and then conduct a quantitative comparative analysis of the policy path of "1+9" water resource tax pilot areas from the perspective of regional heterogeneity.

The analysis establishes two things. The core conditions of all the configuration paths include "water resource endowment," "tax rate design," and "tax collection situation," proving that there is a strong interaction between policy scenario, policy orientation, and policy effect. Therefore, with an in-depth analysis of the policy scenario in the policy field, the enrichment and adjustment in the combination of policy orientations can yield better policy effects; at the same time, by improving the heterogeneity factors that can be artificially regulated in the policy scenario, the overall effect of the policy field can be improved. The typical representative regions of the three configuration paths, Shanxi, Shaanxi, and Sichuan, differ greatly in terms of water resource endowment, industrial structure, and technological level, indicating strong heterogeneity in the implementation paths and effects of water resource tax policies in these pilot areas.

The main contribution of this research is to establish a set of resource tax policy evaluation and comparison methods combining policy process evaluation and quantitative comparison of cases. Meanwhile, it provides reference for China's water resources tax policy adjustment and subsequent expansion of the pilot scope and national promotion. Compared with other studies on water resources tax evaluation, our research does not directly measure the efficiency of water resources tax, but evaluates and compares the water resources tax reforms in each pilot region in China based on the policy field perspective.

This analysis needs to be extended in several ways and a number of limitations apply. Firstly, water resources tax reform is regarded as a holistic policy in this study, but in the real situation the effect of water resources tax reform may be affected by the industry in which it is located. Secondly, regional water use efficiency is selected as the outcome variable in this study, but in reality, the evaluation dimensions and indicators of water resources tax reform effects are diversified. All this is deferred to future research.

**Comment 4: *the number of references are too much! remove unnecessary references.***

**Response 4:**  We appreciate your feedback and agree with your suggestions. We reduced the number of references to 43, and specifically deleted seven references in the original paper, including [22], [24], [27], [28], [32], [37], and [42]. On this basis we have revised the corresponding parts in the original paper and reordered and recoded all the references. For details, please see 'Revised Manuscript with Track Changes'.

**Correction 4:**

Qualitative comparative analysis (QCA) is a research method between case-oriented (qualitative method) and variable-oriented (quantitative method), it is also a comprehensive research strategy that can take advantage of both methods ^[21]^. According to the different set forms, the qualitative comparative analysis method can be subdivided into crisp set qualitative comparative analysis (csQCA), fuzzy set qualitative comparative analysis (fsQCA) and multi-value qualitative comparative analysis (mvQCA) ^[21]^. Because this method can inductively analyze the nature of its configuration in a limited number of cases, it was widely employed in the fields of sociology, economics and management. In the field of resource and environmental policy, the fsQCA methodology has provided scholars with strong methodological support and developed rich results by utilizing its advantage of small-sample case comparison. Specifically, different categories of waste separation policies in 46 pilot cities in China were summarized and compared ^[23]^. Besides, some scholars have applied fsQCA methods to understand how multiple sets of government policies, as captured by public expenditure on climate change issues (environment protection, pollution abatement, waste management) and fiscal strategies (energy, pollution, transport), provide positive or negative ecological footprints ^[25]^.
