# Supplementary material for: Effectiveness evaluation of China’s water resource tax reform pilot and path optimization from the perspective of policy field
Source: PLoS One. 2024 Mar 28;19(3):e0301031. doi: 10.1371/journal.pone.0301031 (PMC10977700; doi:10.1371/journal.pone.0301031)
Supplement: Attachment — Submitted filename: Response to Reviewers0222.docx [file pone.0301031.s004.docx]

**Modification instructions**

Dear editors,

Thanks for providing us with this great opportunity to submit a revised version of our manuscript. We appreciate the detailed and constructive comments provided by the reviewers. We have carefully revised the manuscript by incorporating all the suggestions by the reviewers.

Due to the fact that the affiliation of the first author Yang Mingyi, the School of Humanities and Social Sciences at Beihang University, has been split into the School of Public Administration and the School of Humanities and Social Sciences. The new regulations require that the author affiliation in the academic achievements of students majoring in public administration must be uniformly changed to the School of Public Administration.

We hope this revised manuscript has addressed your concerns, and look forward to hearing from you.

Sincerely,

First author: Mingyi YANG

Second author: Muqi ZHOU

Corresponding author: Conglin ZHANG

**Encl. Responses to the comments from Reviewers.**

**Reply to Reviewer #2**

We appreciate your clear and detailed feedback and hope that the manuscript has fully addressed all of your concerns.Thank you very much for your valuable comments and continued guidance on this paper, which has played a vital role in improving the quality of our paper.
